# Antibacterial Zirconia Surfaces from Organocatalyzed Atom-Transfer Radical Polymerization

**DOI:** 10.3390/ma17081775

**Published:** 2024-04-12

**Authors:** Nesrine Harfouche, Philippe Marie, Diana Dragoe, Hung Le, Pascal Thébault, Christelle Bilot, Arnaud Fouchet, Jacques Rouden, Jérôme Baudoux, Bénédicte Lepoittevin

**Affiliations:** 1 LCMT, UMR 6507, ENSICAEN, UNICAEN, CNRS, Normandie Université, 14000 Caen, Francejerome.baudoux@ensicaen.fr (J.B.); 2 CIMAP, UMR 6252, ENSICAEN, UNICAEN, CNRS, Normandie Université, 14000 Caen, France; 3 ICMMO, UMR 8182, CNRS, Université Paris-Saclay, 91405 Orsay, France; 4 INSA Rouen Normandie, PBS UMR 6270, CNRS, Normandie Université, Université de Rouen Normandie, 76000 Rouen, France; 5 CRISMAT, ENSICAEN, UNICAEN, CNRS, Normandie Université, 14000 Caen, France

**Keywords:** zirconia, cationic polymer, surface modification, surface-initiated photo-ATRP, XPS, water contact angles, antibacterial surfaces

## Abstract

Antibacterial coatings are becoming increasingly attractive for application in the field of biomaterials. In this framework, we developed polymer coating zirconia with antibacterial activity using the “grafting from” methodology. First, 1-(4-vinylbenzyl)-3-butylimidazolium chloride monomer was synthesized. Then, the surface modification of zirconia substrates was performed with this monomer via surface-initiated photo atom transfer radical polymerization for antibacterial activity. X-ray photoelectron spectroscopy, ellipsometry, static contact angle measurements, and an atomic force microscope were used to characterize the films for each step of the surface modification. The results revealed that cationic polymers could be successfully deposited on the zirconia surfaces, and the thickness of the grafted layer steadily increased with polymerization time. Finally, the antibacterial adhesion test was used to evaluate the antibacterial activity of the modified zirconia substrates, and we successfully showed the antibacterial activity against *Staphylococcus aureus* and *Pseudomonas aeruginosa* strains.

## 1. Introduction

Zirconia (ZrO_2_), a crystalline oxide of zirconium, has emerged as a promising and appropriate ceramic biomaterial for implant manufacturing, and it is one of the most important alternatives to titanium and titanium alloys [1,2]. Due to aesthetic advantages, such as color and opacity that imitate natural teeth appearance, dense zirconia or yttria-stabilized tetragonal zirconia polycrystal (Y-TZP) has been widely employed as multi-unit bridges, crowns, onlays, inlays, and implant structures [3]. Zirconia-based materials have been touted as a biomaterial with high chemical stability that prevents hazardous chemicals from being released into the surrounding tissues [4]. However, the bio-inert nature of zirconia could restrain its use as a biomedical implant. Surface modification can be applied to customize the pattern surface and overcome zirconia’s bio-inert nature [5,6].

To date, implant technology has made great efforts to modify zirconia in terms of morphological and bioactive features that are desirable for cell attachment, proliferation, and differentiation during surrounding bone healing [7]. Even though the development of a functionally graded material for implants has demonstrated an improvement in biological response and mechanical behavior, the development of coatings composed of graphene, silica, magnesium, dopamine, and bioactive compounds has been explored. Surface modification via polymer grafting is considered to be an effective approach to prevent the accumulation of organisms on surfaces and the development of infections [8,9,10]. 

From this perspective, several antifouling and antibacterial functional polymers, such as cationic polymers, zwitterionic polymers, hydrophilic or amphiphilic polymers, silicone, or fluoropolymers, have been coated on the surface of various solid materials [11,12]. Among these functional polymers, cationic polymers are widely used as antifouling and antibacterial coatings [13].

Cationic polymers have gained more interest than biocides based on small molecule cationic compounds due to their reduced toxicity and improved antimicrobial activities. Recently, quaternary ammonium [14], pyridinium [15], piperidinium, pyrrolidinium [16], phosphonium [13], and imidazolium [17] cation-based antimicrobial polymers were synthesized and used for antimicrobial applications. 

**Figure 1:**
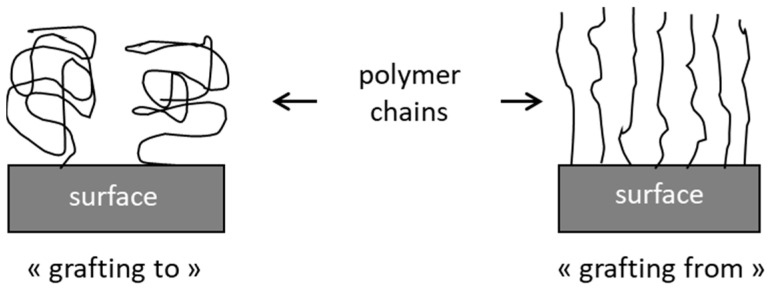
Schematic representation of “grafting to” and “grafting from” strategies.

The anchoring of cationic polymers by a “grafting-to” technique or growing polymer chains from a surface-bound initiator by a “grafting-from” technique have been employed to covalently modify a variety of surfaces to make them bactericidal and/or resistant to bacterial adherence [18] (Figure 1). The “grafting to” technique allows the grafting of a preformed polymer with a surface bearing a reactive functional group. Due to steric hindrance, the grafting density is low (mushroom). The “grafting from” strategy is more appealing due to its high-tethering density with a homogenous polymer coating on the solid surface, which is accompanied by high stability. In this approach, the polymerization is directly initiated from initiator-anchored surfaces, and initiator density can be relatively high [19,20].

Polymer brushes could be grafted onto a variety of solid surfaces via the “grafting from” technique, which is often based on reversible deactivation radical polymerizations [21,22]. Atom transfer radical polymerization (ATRP) has proven to be a useful and adaptable tool for building well-defined polymer brushes with molar masses and graft density controls [23,24]. The main advantages of ATRP are its tolerance to a wide range of function groups on the monomer and the possibility to polymerize at room temperature in a large variety of solvents [25,26]. In addition, a suitable alkyl halide initiator group can be easily introduced via a reaction with 2-bromoisobutyryl bromide with surfaces bearing hydroxyl groups. Nevertheless, one of the ATRP’s major drawbacks is that it requires a substantial amount of the toxic transition metal catalyst, which is not suitable for biological systems. Another key point that makes the ATRP reaction even more difficult is the oxygen sensitivity of metal complexes and the need for a completely inert environment. The above disadvantages can be overcome using photochemical ATRP methods. These techniques allow temporal and spatial control of the processes because of their simple experimental setup, negligible side effects and flexible light source [27,28].

Metal-free photo-induced ATRP is an organic photoredox system reduced in the excited state that avoids the metal contamination problems that plague regular ATRP. Additionally, the metal-free ATRP method enables the regeneration of the catalytic complex by incorporating an organic photoredox catalyst under light irradiation [29,30,31].

Recent research has shown that photoinduced ATRP can be performed using a conventional Type II photoinitiator (e.g., eosin Y, fluorescein, phycoerythrin B and camphorquinone) in conjunction with co-initiators and alkyl halides as initiators [32]. 

As part of our recent efforts to develop new methods for zirconia surface modification [33,34], we now report the grafting of poly(1-(4-vinylbenzyl)-3-butylimidazolium chloride) by photo-induced metal-free ATRP for antibacterial applications. Camphorquinone, as a Type II photoinitiator, was used for the photo-induced ATRP polymerization in combination with PMDETA (*N*,*N*,*N*′,*N*′,*N*″,*N*″-pentamethyldiethylenetriamine) as an electron donor source. 

## 2. Experimental Section

### 2.1. Materials

4-vinylbenzyl chloride (CAS 1592-20-7, Sigma-Aldrich, St. Louis, MO, USA, 90%), 1-(N-butyl)imidazole (CAS 4316-42-1, Alfa Aesar, Haverhill, MA, USA, 99%), 2-dimethylaminopyridine (DMAP) (CAS 5683-33-0, TCI, 99%), triethylamine (CAS 121-44-8, Acros, Geel, Belgium, 99%), tetrahydrofuran (THF, CAS 109-99-9), 2-bromoisobutyryl bromide (BiBB) (CAS 20769-85-1, Alfa Aesar, 97%), ethyl 2-bromoisobutyrate (EBiB) (CAS 600-00-0, Sigma-Aldrich, 98%), *N*,*N*,*N*′,*N*′,*N*″,*N*″-pentamethyldiethylenetriamine (PMDETA) (CAS 3030-47-5, Aldrich 99%), *N*,*N*-dimethylformamide (DMF) (CAS 68-12-2, Fisher Chemical, Hampton, NH, USA), methanol (CAS 67-56-1, VWR chemicals, Solon, OH, USA) and camphorquinone (CQ) (CAS 10373-78-1, Alfa Aesar, 99%) were used as received. Diiodomethane (CAS 75-11-6, Thermo Scientific, Waltham, MA, USA) and formamide (CAS 75-12-7, Sigma-Aldrich, 99%) were used for contact angle measurements. H_2_SO_4_ (CAS 7664-93-9, ACROS ORGANICS, 96%) and H_2_O_2_ (CAS 7722-84-1, VWR chemicals, 30 wt% in H_2_O) were used for piranha treatment. Photopolymerization reactions were made in 20 mL screw vials with 24 mm of clear glass from Interchim (CK1540) using an Evolu Chem Photo RedOx Box Hepato Chem with a blue led lamp (450–455 nm) purchased from Interchim (Montluçon, France).

### 2.2. Preparation of Yttria Tetragonal Zirconia Polycrystal (Y-TZP) Substrates

The Y-TZP samples were obtained from a Tosoh TZ-3YB-E zirconia ZrO_2_ powder (Amsterdam, The Netherlands) with 3 mol% Y_2_O_3_ (particle size 40 nm). A quantity of 1.5 g of the powder was introduced into a 1.6 cm diameter mold and pressed at 100 MPa. The Y-TZP disks were placed in an oven and sintered at 1500 °C for 2 h. The heating and cooling ramps were 150 °C/h. After cooling to room temperature, we obtained yttria zirconia disks (diameter: 13 mm, thickness: 3 mm). Then, Y-TZP disks were polished in the following two steps: 1/mechanically with abrasive papers (220-grit, 500-grit and 1200-grit) and water, 2/the disks were polished with Largo, DAC and NAP abrasive papers and diamond solutions to obtain Y-TZP disks. Finally, the Y-TZP disks were ultrasonically cleaned in acetone and then dried for a few minutes under an argon atmosphere. Before grafting, the Y-TZP disks were cleaned and activated by freshly prepared “piranha” (98% H_2_SO_4_/30% wt H_2_O_2_ in H_2_O; 70/30 *v*/*v*) for 1 h at room temperature and rinsed thoroughly with deionized water until they reached a neutral pH (Y-TZP-OH surfaces) [34].

### 2.3. Immobilization of ATRP Initiators on the Zirconia Substrates

The zirconia substrate was immersed in 10 mL of dry THF solution containing triethylamine (0.54 mL, 3.9 × 10^−3^ mol) and a catalytic amount of DMAP (0.04 g, 3.2 × 10^−4^ mol). The mixture was placed under an ice bath, and BiBB (0.4 mL, 3.2 × 10^−3^ mol) was then added into the mixture dropwise. The reaction proceeded at 20 °C for 24 h. Then, the functionalized zirconia-based initiator pellets (hereinafter referred to as Y-TZP-Br) were removed from the solution and carefully rinsed with dichloromethane and ethanol and dried under an argon atmosphere.

### 2.4. Surface-Initiated Photo-ATRP from Y-TZP-Br Substrates

Y-TZP-Br pellets were placed into dry flasks under nitrogen. A solution (DMF, 5 mL) containing an ionic liquid monomer (1.38 g, 4.9 mmol, 100 eq), PMDETA (50 µL, 43 mg, 0.24 mmol, 5 eq), camphorquinone (8.3 mg, 0.05 mmol, 1 eq), and EBiB (7 µL, 9.7 mg, 0.047 mmol, 1 eq) were then added into each flask under an argon atmosphere. The reaction medium was irradiated using a photoreactor, which was equipped with a lamp emitting light of λ = 450 nm at room temperature. The functionalized zirconia surfaces (hereinafter referred to as Y-TZP-PIL) were removed from the solution at different times of polymerization, carefully washed with methanol to remove the ungrafted polymer, and dried under a vacuum. 

### 2.5. Characterizations

Monomer conversion was determined by ^1^H NMR (Bruker Avance III 500 MHz, Billerica, MA, USA) and an integration comparison between the residual peaks of the monomer and the peaks of the polymer. An example of ^1^H spectra showing the evolution of monomer conversion is presented in Figure S1. Samples were dissolved in D_2_O.

X-ray photoelectron spectroscopy (XPS) measurements were performed on a K-Alpha spectrometer from ThermoFisher, equipped with a monochromated X-ray source (Al Kα, 1486.6 eV). For all measurements, a spot size of 400 µm was employed, corresponding to an irradiated zone of ~1 mm^2^. The hemispherical analyzer was operated in the CAE (Constant Analyzer Energy) mode, with a pass energy of 200 eV, a step of 1 eV for the acquisition of the survey’s spectra, a pass energy of 50 eV and a step of 0.1 eV for the acquisition of narrow scan spectra. A “dual beam” flood gun with low-energy electrons and argon ions was used to compensate for surface charging. The recorded spectra were processed by means of the Avantage software (https://www.thermofisher.com/), using a peak-fitting routine with Shirley background and symmetrical 30–70%-mixed Gaussian–Lorentzian line shapes. Atomic percentages were calculated following normalizations of peak areas using the Scofield sensitivity factors. The fitting procedure consisted of adding identical synthetic line shapes with similar widths and corresponding to components derived from the chemical structure of the grafted molecule correlated with the knowledge of chemical shift from databases and the literature. Additional constraints were added concerning the BE and the area ratio of different components. In order to check the quality of fit, normalized areas of corresponding components from all core-level spectra were compared and found to be in the limit of precision for XPS (5–15%). The binding energy scale was calibrated against neutral carbon considered at 285 eV. The precision in binding energy was ±0.2 eV. Four different areas at the sample surface were measured in order to check the homogeneity of the organic grafted layer.

For contact angle measurements, a DSA30 Krüss goniometer (Krüss, Hamburg Germany) was used with the Young–Laplace method. The static contact angles were measured within 5 s of placing the drop (1 μL) on the surfaces, and an average of five measurements was calculated. High-purity water (Millipore, Burlington, MA, USA, milliQ) was used.

The surface free energy values of Y-TZP samples were determined by measuring the contact angle of the three test liquids. Formamide (CH_3_NO) and diiodomethane (CH_2_I_2_) were used as a polar and nonpolar solvent, respectively. The approach of Good, Van Oss and Chaudhury (the acid-based theory) was used to calculate the surface free energy and energetic characteristics [35].

The thickness of organic coating layers on the zirconia surface was measured with a spectroscopic ellipsometer. Spectroscopic ellipsometry measurements were carried out using a Jobin Yvon UVISEL2 (HORIBA, Kyoto, Japan) setup at an incidence angle of 70°. The spectra were recorded over a 1.5–6 eV range with 0.05 eV resolution. The refractive indices, as well as the thicknesses, were deduced from the experimental data using a dispersion law in DeltaPsi2 software (https://www.horiba.com/int/products/detail/action/show/Product/deltapsi2-software-1648). The corresponding optical dispersion is a single oscillator derived from the Forouhi–Bloomer model, which is applicable to dielectric materials [36].

The surface topography and the roughness of the samples were examined using atomic force microscopy (AFM) from Molecular Imaging (Pico SPM-LE, Meadowbank, NSW, Australia) in the tapping mode. The roughness profile provided peak-to-valley surface roughness (Ra) measurements.

Scanning electron microscopy (SEM JEOL JSM 7200F, JEOL Ltd., Tokyo, Japan) equipped with an energy-dispersive spectroscopy (EDS Bruker XFlash 6-100, Bruker, Billerica, MA, USA) system was used to examine the surface morphologies of the zirconia Y-TZP-OH and zirconia-modified pellets (Y-TZP-PIL). At a 5 kV accelerating voltage, SEM pictures were taken.

### 2.6. Measurements of the Antibacterial Activities

(a) Bacterial culture

All materials were autoclaved at 121 °C for 15 min. Bacteria were kept at −20 °C in a 30% glycerol solution. *Staphylococcus aureus* ATCC 29213 and *Pseudomonas aeruginosa* PA14 strains were precultured under aerobic conditions in a “Brain Heart Infusion” nutritive medium (BHI, Bacto™, Mount Pritchard, NSW, Australia) at 37 °C and 140 rpm for 24 h. The bacterial suspension was diluted in PBS medium to achieve a 0.01 optical density at 600 nm, corresponding to a bacterial concentration around 10^6^ CFU.mL^−1^. This bacterial concentration was confirmed by counting on BHI agar (Difco™, Franklin Lakes, NJ, USA).

(b) Bacterial adhesion test

Zirconia and modified zirconia pellets were immersed in 2 mL bacterial suspensions at 10^6^ CFU.mL^−1^ into wells of 24P and incubated at 37 °C under static conditions. After 3 h, the substrates were rinsed with a sterile Milli-Q water solution to eliminate non-adhered bacteria. These substrates were immersed in 4 mL of a PBS buffer solution and sonicated for 5 min. Decimal dilutions, i.e., 10^−0^, 10^−1^, 10^−2^ and 10^−3^ of the bacterial suspension were spread onto BHI agar plates. After incubation for 24 h at 37 °C, the colonies were enumerated and expressed as CFU⋅cm^−2^. Three independent enumerations were carried out for each assay, which was performed in triplicate.

The inhibition values were expressed as percentages compared to the Y-TZP-OH surface as a control.

(c) Confocal microscopy analysis

Adhered bacteria on the substrates were also visualized by confocal microscopy. The Filmtracer LIVE/DEAD Biofilm Viability Kit (Molecular Probes-Invitrogen, Santa Clara, CA, USA) was used to assess the membrane integrity of the adhered bacteria. For labeling, Syto 9 (3.34 mM) and propidium iodide (20 mM) were mixed in equal proportions, diluted 1000 times, and 150 µL of the resulting solution was deposited onto surfaces. After 30 min of incubation in the dark, surfaces were rinsed with a sterile PBS buffer solution and mounted on a microscope slide with a mowiol^®^ 4–88 solution (Polysciences, Inc., Warrington, PA, USA). Surfaces were observed using a confocal microscope Leica TCS SP8 CFS (Leica Microsystems, Nanterre, France). The excitation of Syto9 and propidium iodide were performed at 488 nm and 552 nm, respectively. Fluorescence emission was detected sequentially by a hybrid detector (Leica Microsystems, Nanterre, France) in the photon counting mode with a specific band from 500 to 540 nm for Syto9 and from 580 to 630 nm for propidium iodide. Image processing was performed with Imaris software (Bitplane©, Schlieren, Switzerland, https://imaris.oxinst.com/).

## 3. Results and Discussion

### 3.1. Preparation of Zirconia Pellets, Ionic Liquid Monomer Synthesis and Initiator Grafting

In order to obtain antibacterial zirconia surface-by-polymer grafting, the ionic liquid monomer 1-butyl-3-[(4-vinylbenzyl]-imidazolium chloride (IL_sty_) was chosen due to the antibacterial activity of poly(ionic liquid)s polymers known in the literature [17,37,38]. This monomer was obtained in one step on a large scale by the *N*-alkylation of *N*-butylimidazole by 4-vinylbenzyl chloride according to well-established protocols [19,39].

**Scheme 1:**
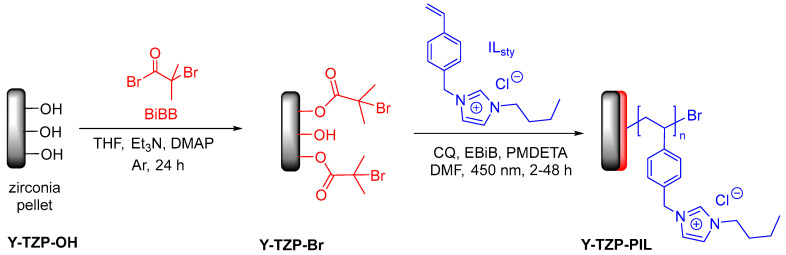
Grafting of initiator (first step) and surface-initiated ATRP (second step) from Y-TZP-OH.

Simultaneously, zirconia ceramic pellets (Y-TZP for tetragonal zirconia polycrystal) were obtained from 97 mol% ZrO_2_ stabilized by 3 mol% Y_2_O_3_ by sintering at 1500 °C for two hours, followed by polishing. Then, they were treated with a piranha solution in order to remove organic residues from polishing and to oxidize the surface by introducing hydroxyl groups (Y-TZP-OH), as we previously showed [33,34]. Then, in order to perform ATRP polymerization, zirconia pellets were reacted with 2-bromoisobutyryl bromide (BiBB) to introduce bromo alkyl groups by the esterification reaction (Scheme 1, step 1). This reaction was carried out with an excess of BiBB in THF with triethylamine and DMAP. Surfaces were thoroughly washed (dichloromethane and ethanol) to remove unreacted species.

In order to confirm the initiator grafting, contact angle measurements with different polar (water and formamide) and apolar (diiodomethane) liquids were performed, and the surface energy values were calculated from the Young–van Oss equation [33,35]. Values are given in Table 1.

The water contact angles of the native zirconia surface (Y-TZP) and surface obtained after piranha treatment (Y-TZP-OH) are equal to 60 and 21°, respectively. These values confirm the treatment effect by removing organic contamination and the formation of hydroxyl groups [34]. After the reaction with 2-bromoisobutyryl bromide (Y-TZP-Br surface), the water contact angle increased from 21 to 59°, indicating a reduction in hydrophilicity by covering the surface with alkyl bromide groups. The total surface energy of native Y-TZP and piranha-treated Y-TZP-OH zirconia surfaces were equal to 40 and 57 mJ·m^−2^, respectively. ATRP initiator grafting results in lower total surface energy (46.81 mJ·m^−2^ for Y-TZP-Br) with a decrease in the electron donor δ-(45.86 mJ·m^−2^ for Y-TZP-OH and 19.23 mJ·m^−2^ for Y-TZP-Br) due to the disappearance of hydroxyl groups on the surface. The surface energy characteristics (δ_s_, δ^LW^, δ^+^, and δ^−^) are in very close agreement with the values obtained for the chitosan surfaces treated with 2-bromoisobutyryl bromide, confirming the success of the initiator grafting [24].

### 3.2. Ionic Liquid Monomer Polymerization Initiated from the Zirconia Surface

The ATRP of the IL_sty_ monomer was carried out under light irradiation from grafted initiators in the presence of a sacrificial initiator (EBiB) in order to control molar masses. Camphorquinone (CQ) was chosen as a photosensibilizer, together with PMDETA as the electron donor source. It was previously shown by Yagci et al. that camphorquinone is a Type II photoinitiator for UV-visible-induced electron transfer processes (with the irradiation wavelength in the range of 400–500 nm) [32]. Additionally, the same study showed that polymerizations in polar solvents (such as DMF and DMSO) give higher conversions and polymers with narrow molar mass distributions.

According to this study, the “grafting from” polymerization of the IL_sty_ monomer was performed in a DMF solution with EBiB as the sacrificial initiator and PMDETA and camphorquinone as the catalytic system (Scheme 1) with a molar ratio [monomer]/[EBiB]/[PMDETA]/[CQ] equal to 100/1/5/1. Polymerizations were activated under visible light irradiation (450 nm). The evolutions of the water contact angles and the film thickness as the reaction time are presented in Table 2.

Depending on the polymerization time, the water contact angle decreased from 59° to 35° and remained constant after 8 h of the reaction. Hydrophilicity increased after polymerization due to the presence of imidazolium heterocycles on the surface. Then, polymerized ionic liquid resulted in a more hydrophilic surface than Y-TZP-Br with an increase in the total surface energy from 46.8 to 50.9 mN/m, respectively, and a significant increase in the electron donor δ^−^ component (from 19.23 to 41.03 mN/m) due to the presence of nitrogenous molecules on the surface (Table 1).

**Figure 2:**
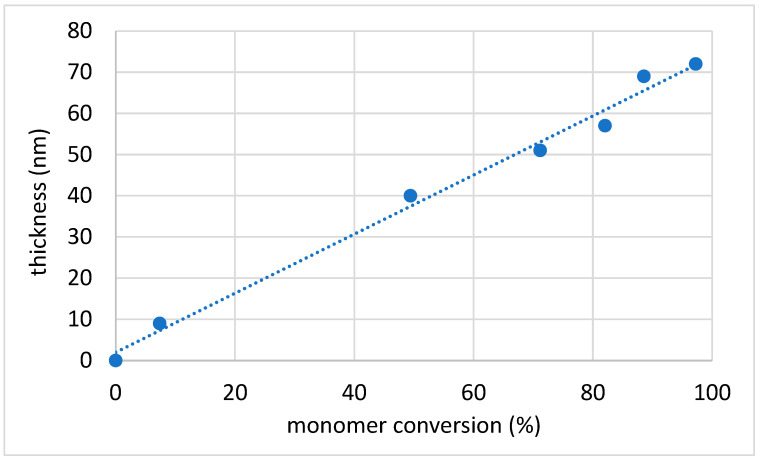
Dependence of the polymer thickness of the Y-TZP-PIL (determined by ellipsometry) on monomer conversion (determined by ^1^H NMR).

The evolution of the polymer thickness on the zirconia surfaces (Y-TZP-PIL) was determined by ^1^H NMR and is shown in Figure 2. The thickness of the grafted polymer layer increased linearly with monomer conversion to reach a value of around 70 nm for 96% monomer conversion. This linear relationship indicated that the photo-induced polymerization process was under control, as previously shown in the literature for the classical ATRP “grafting from” polymerization [40]. To the best of our knowledge, this behavior has not yet been reported in the literature for metal-free ATRP under UV-visible light irradiation with camphorquinone as the sensitizer.

The surface composition variations in the native zirconia (Y-TZP-OH) and modified zirconia by polymer grafting (Y-TZP-PIL) were characterized by XPS using the atomic detection of C, O, Y, Zr, N, Cl and Br. Table 3 lists the detailed data from XPS scans on the two different surfaces.

**Figure 3:**
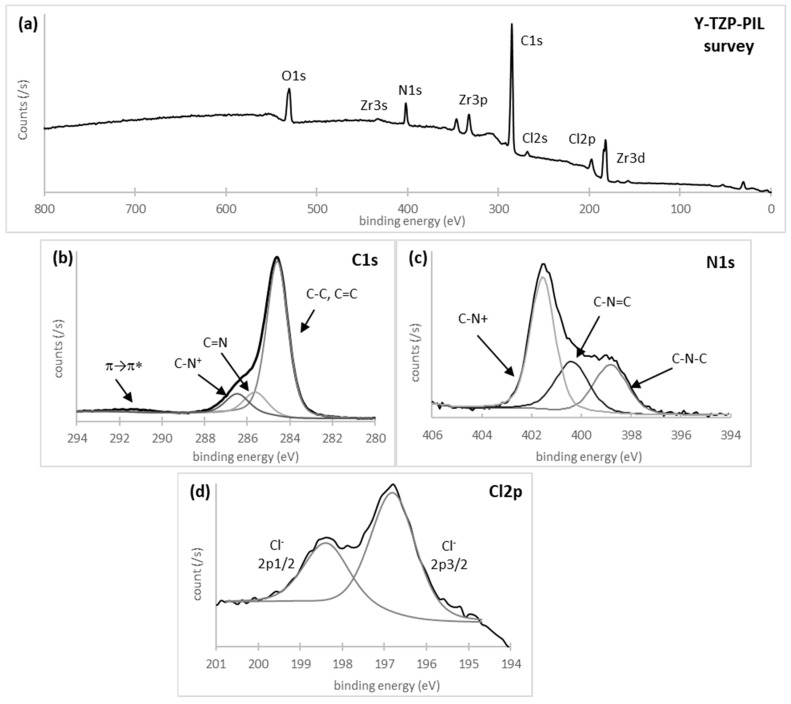
XPS characterization of Y-TZP-PIL surface (obtained after 4 h of polymerization time): a survey (**a**), core-level spectrum of C1s (**b**), N1s (**c**), and Cl2p (**d**): high-resolution spectra (black) and decomposition and curve-fitted spectra (shade of gray).

After piranha treatment (Y-TZP-OH surface), the atomic composition of zirconium and oxygen was equal to 29.9% and 59.7%, respectively. Thus, the atomic ratio Zr/O was close to ½ the theoretical value for zirconia (ZrO_2_). In total, 1.8% of yttria was presented as coming from yttria (Y_2_O_3_), which is used as a stabilizer in ceramic technology. The atomic concentration of carbon (8.6%) was due to some residual adsorbed species, mainly hydrocarbons. In Figure 3, the XPS survey (Figure 3a) of the surface obtained after ionic liquid monomer polymerization and the high-resolution spectra of C1s, N1s, and Cl2p are presented (Figure 3b, Figure 3c and Figure 3d, respectively).

The C1s core-level spectrum after ionic liquid monomer polymerization can be fitted with four carbon contributions (Figure 3b): a main peak for C-C/C=C (284.6 eV), and three less intense peaks with binding energies equal to 291.9, 286.5, and 285.7 eV assigned to the shake-up of π electrons, C-N^+^ and C=N, respectively. After this polymerization, a new peak appeared around 400 eV corresponding to the N1s of imidazolium heterocycle on the monomer unit (with the atomic percentage equal to 6%) (Table 3). The N1s core-level spectrum was curve-fitted with three components at 401.6, 400.4, and 398.9 eV assigned to C-N^+^, C=N, and C-N, respectively (Figure 3c). In addition, a new contribution corresponding to Cl2p (chlorine ion associated with imidazolium cation) appeared in the range of 200 to 195 eV. The Cl2p core-level spectrum showed that chlorine was present in the following different states: Cl 2p_1/2_ at 198.4 eV and Cl 2p_3/2_ at 196.8 eV (Figure 3d). Some residual peaks coming from zirconia sublayers were visible. In conclusion, the XPS results combined with ellipsometry measurements proved the presence of a polymer organic layer on the zirconia surface.

**Figure 4:**
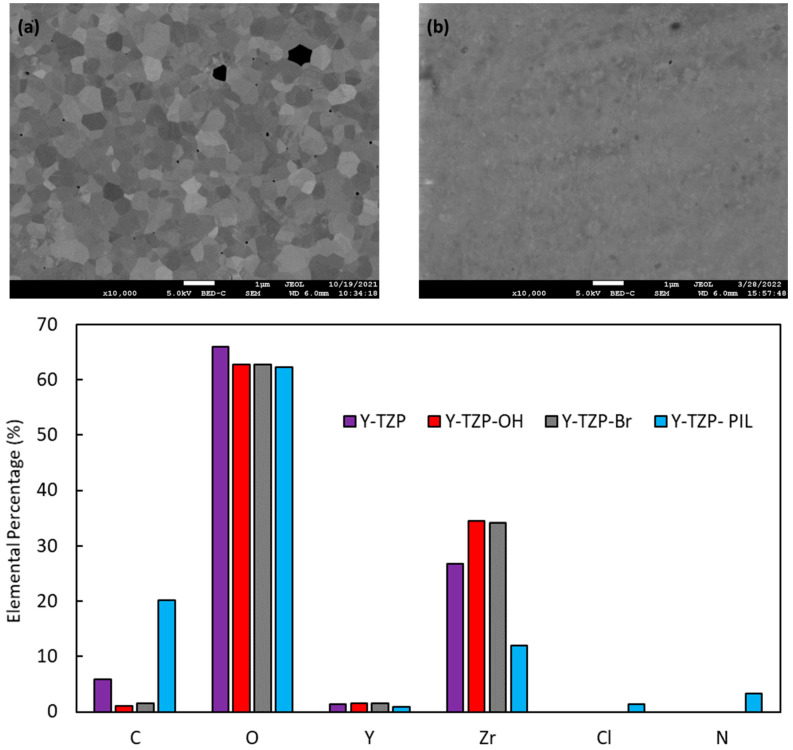
Surface morphology images obtained by the SEM of (**a**) Y-TZP-OH, (**b**) Y-TZP-PIL (scale: 1 µm), and the EDS analysis (elemental percentage) of Y-TZP, Y-TZP-OH, Y-TZP-Br, and Y-TZP-PIL.

Microscopy characterizations (SEM and AFM) were performed to study the evolution of surface morphology after polymerization. SEM images at 10,000× magnification of the Y-TPZ-OH and Y-TZP-PIL are shown in Figure 4. SEM was further used to check the presence of the organic layer on the surface. The image of the surface grafted with polymer film (Figure 4b) was detected by a very thin layer and compared with that of the Y-TPZ-OH substrate (Figure 4a), where substrate grains were observed. The images exhibited a smooth surface for both substrates. The coating surface was smooth and homogeneous, and this homogeneity conferred to the film’s excellent transparency. Energy dispersive X-ray spectroscopy (EDS) elemental analysis was carried out to understand the success of each step of the procedure (Figure 4). A decrease in the carbon content was observed on the Y-TZP-OH substrate compared to Y-TZP, which confirmed a good cleaning of zirconia pellets after piranha treatment. After initiator grafting, the thickness of the organic layer containing the atom bromide was too thin for the EDS detector to identify it, or the bromide concentration was too low. The EDS analysis of Y-TZP-PIL indicated the existence of chlorine and nitrogen species, suggesting that imidazolium was successfully grafted onto the zirconia surface.

**Figure 5:**
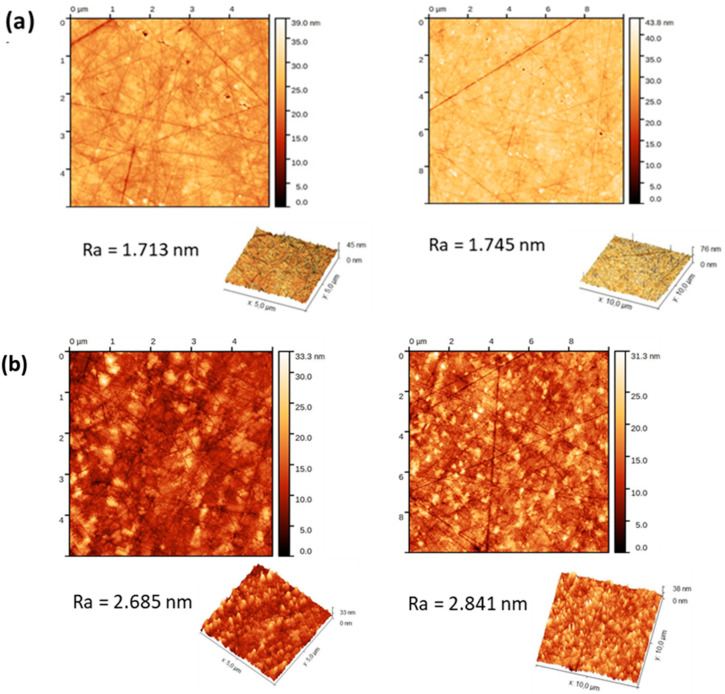
AFM topography and 3D image of (**a**) Y-TZP-OH and (**b**) Y-TZP-PIL surfaces.

AFM analyses revealed topographical changes in the zirconia surfaces after the photopolymerization of the cationic monomer (Figure 5). The native zirconia substrate exhibited a very smooth surface with an arithmetic roughness of around 1.7 nm (Figure 5a). After polymerization, the roughness of the Y-TZP-PIL surface increased slightly and was around 2.8 nm (Figure 5b). In the topography images (Figure 5b), small domains are visible. This observation could be explained by polymer dimples on the top of the surface [41]. However, the homogeneous grafting of the ionic polymer was observed on the surface. 

### 3.3. Antibacterial Study

The antibacterial activity of the Y-TZP-PIL surface was evaluated after 3 h of contact with bacterial cells by counting adhered bacteria and fluorescence microscopy visualization. The adhesion of *S. aureus* and *P. aeruginosa* was studied due to their membership in the ESKAPE group [42].

All antibacterial activities were normalized based on cultivable bacterial adhesion on Y-TZP-OH surfaces and expressed as a percentage. As observed in Table 4, a decrease in the bacterial adhesion of both species was observed. Indeed, bacterial adhesion inhibition of 93.2 and 96.5% were observed, respectively, for *S. aureus* and *P. aeruginosa*.

**Figure 6:**
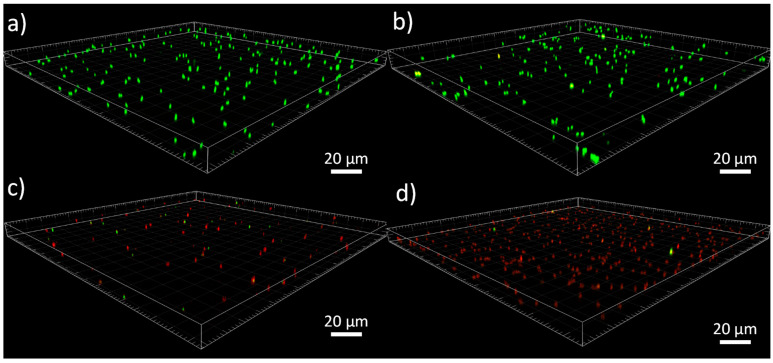
Confocal fluorescence images of Y-TZP-OH (**a**,**b**) and Y-TZP-PIL (**c**,**d**) zirconia surfaces after 3 h of contact with a suspension of *S. aureus* and *P. aeruginosa*, respectively. Bacteria were stained using the Filmtracer LIVE/DEAD Biofilm Viability Kit, Molecular Probes-Invitrogen.

The antibacterial mechanism of imidazolium-based ionic liquids is known to begin with electrostatic interactions between the positive charges of the heterocyclic units and the negative charges of the bacterial wall. Then, the hydrophobic chains of the imidazolium insert into the bacterial membrane, leading to its destabilization, pore formation, and eventual bacterial death [17]. To verify if the decrease in bacterial adhesion comes from the imidazolium groups, the membrane integrity of adhered bacteria was studied by confocal fluorescence microscopy. Bacterial cells with intact membranes were indicated by green coloration (Syto9 stain), whereas cells with damaged membranes exhibited red coloration (propidium iodide stain). Representative images of the adhered bacteria on Y-TZP-OH and Y-TZP-PIL surfaces are shown in Figure 6. For Y-TZP-OH surfaces, only green bacteria can be observed, indicating the absence of antibacterial activity on such surfaces. Conversely, on modified zirconia surfaces, the majority of adhered bacteria, whatever the species, appear in red, confirming the previously discussed mechanism of antibacterial imidazolium moieties.

## 4. Conclusions

In this study, we report a new strategy to modify the zirconia surface by the “grafting from” polymerization of an ionic styrenic monomer bearing an imidazolium heterocycle. This monomer was prepared in one step on a large scale by *N*-alkylation. Surface photo-induced metal-free atom transfer radical polymerization was performed on the zirconia surface using camphorquinone as a photoinitiator under visible light activation at 450 nm and in the presence of a sacrificial initiator. A good control of polymerization was obtained, as attested by the linear evolution of organic coating thickness according to the monomer conversion detected by ^1^H NMR. At the end of polymerization, a coating of 70 nm thickness was measured by ellipsometry. In addition, a full surface characterization was performed by a combination of different techniques, including contact angle measurements, X-ray photoelectron spectroscopy, and microscopies. The zirconia substrates grafted with the ionic liquid polymer were found to exhibit good antibacterial activity against *Staphylococcus aureus* and *Pseudomonas aeruginosa* strains, with the inhibition of bacterial adhesion inhibition being 93.2 and 96.5%, respectively. The surface treatment proposed in this study improves the biological performance of zirconia when used as a dental biomaterial.

## Figures and Tables

**Table 1:** Calculated values of solid surface energy δ_S_, the Lifshitz-van der Waals component δ^LW^, the electron acceptor δ^+^ and the electron donor δ^−^ parameters of the acid-base component of surfaces and contact angles values with different liquids.

Surface	Surface Energy Characteristics (mJ·m^−2^)				Contact Angle (°)		
	δ_S_	δ^LW^	δ^+^	δ^−^	Water	CH_2_I_2_	CH_3_NO
Y-TZP ^a^	40.32	34.89	0.32	23.05	59.9	48.9	49.8
Y-TZP-OH ^a^	57.87	36.20	2.56	45.86	21.1	48	4.4
Y-TZP-Br	46.81	35.31	1.72	19.23	58.8	48.1	37.5
Y-TZP-PIL ^b^	50.92	37.90	1.03	41.03	35.0	43.3	28.2

^a^ Adapted from [34]. ^b^ Values obtained with a polymerization time equal to 4 h.

**Table 2:** Evolution of static water contact angle and film thickness determined by ellipsometry according to the different steps.

Surface	Polymerization Reaction Time (h)	Film Thickness (nm)	Static Water Contact Angle (deg)
Y-TZP	-	-	60 (±3)
Y-TZP-OH	-	-	21 (±6)
Y-TZP-Br	-	2	59 (±2)
Y-TZP-PIL	4	9	37 (±1)
Y-TZP-PIL	8	40	35 (±1)
Y-TZP-PIL	12	51	35 (±2)
Y-TZP-PIL	18	57	35 (±2)
Y-TZP-PIL	24	69	35 (±2)
Y-TZP-PIL	48	72	35(±2)
Y-TZP-OH ^a^	24	1	24 (±3)

^a^ A control experiment was performed using the Y-TZP-OH substrate instead of Y-TZP-Br. In this case, no grafted polymer was observed, as attested by the water contact angle and the ellipsometry measurements.

**Table 3:** Elemental surface composition of zirconia substrates determined from XPS.

	Experimental Atomic Ratio %						
Surfaces	Zr3d	Y3d	O1s	C1s	Br3d	N1s	Cl2p
Y-TZP-OH	29.9	1.8	59.7	8.6	-	-	-
Y-TZP-PIL ^a^	6.1	<1	12.1	73.2	<1	6.1	2.0

^a^ Values obtained with a polymerization time equal to 4 h.

**Table 4:** Bacterial adhesion inhibition of the Y-TZP-PIL surface. The inhibition values (%) are given compared to the Y-TZP-OH surface. Values are the means of three independent experiments (n = 3).

Surface	*S. aureus*		*P. aeruginosa*	
	Number of Adhered Bacteria	Bacterial Adhesion Inhibition	Number of Adhered Bacteria	Bacterial Adhesion Inhibition
Y-TZP-OH	4.20 × 10^5^ CFU.cm^−2^	-	1.4 × 10^5^ CFU.cm^−2^	-
Y-TZP-PIL (40 nm)	2.85 × 10^4^ CFU.cm^−2^	93.2%	4.9 × 10^3^ CFU.cm^−2^	96.5%

## Data Availability

Data are contained within the article or as Supplementary Materials.

## Supplementary Materials

The following supporting information can be downloaded at: https://www.mdpi.com/article/10.3390/ma17081775/s1, Figure S1: Evolution of ^1^H NMR spectra (Bruker Avance III 500 MHz) during polymerization. Samples were dissolved in D_2_O.
