# Supplementary material for: Antibacterial Zirconia Surfaces from Organocatalyzed Atom-Transfer Radical Polymerization
Source: Materials (Basel). 2024 Apr 12;17(8):1775. doi: 10.3390/ma17081775 (PMC11051261; doi:10.3390/ma17081775)
Supplement: Supplementary file 1 [file materials-17-01775-s001.zip › materials-2950017-supplementary.pdf]

Article

Antibacterial Zirconia Surfaces from Organocatalyzed Atom-Transfer Radical Polymerization

Nesrine Harfouche [1], Philippe Marie [2], Diana Dragoe [3], Hung Le [4], Pascal Thébault [4], Christelle Bilot [5], Arnaud Fouchet [5], Jacques Rouden [1], Jérôme Baudoux [1] and Bénédicte Lepoittevin [1,*]

1 LCMT, UMR 6507, ENSICAEN, UNICAEN, CNRS, Normandie Université, 14000 Caen, France; nesrine.harfouche@ensicaen.fr (N.H.); jacques.rouden@ensicaen.fr (J.R.); jerome.baudoux@ensicaen.fr (J.B.)
2 CIMAP, UMR 6252, ENSICAEN, UNICAEN, CNRS, Normandie Université, 14000 Caen, France; philippe.marie@ensicaen.fr
3 ICMMO, UMR 8182, CNRS, Université Paris-Saclay, 91405 Orsay, France; diana.dragoe@universite-paris-saclay.fr
4 INSA Rouen Normandie, PBS UMR 6270, CNRS, Normandie Université, Université de Rouen Normandie, 76000 Rouen, France; hung.le@univ-rouen.fr (H.L.); pascal.thebault@univ-rouen.fr (P.T.)
5 CRISMAT, ENSICAEN, UNICAEN, CNRS, Normandie Université, 14000 Caen, France; christelle.bilot@ensicaen.fr (C.B.); arnaud.fouchet@ensicaen.fr (A.F.)
* Correspondence: benedicte.lepoittevin@ensicaen.fr

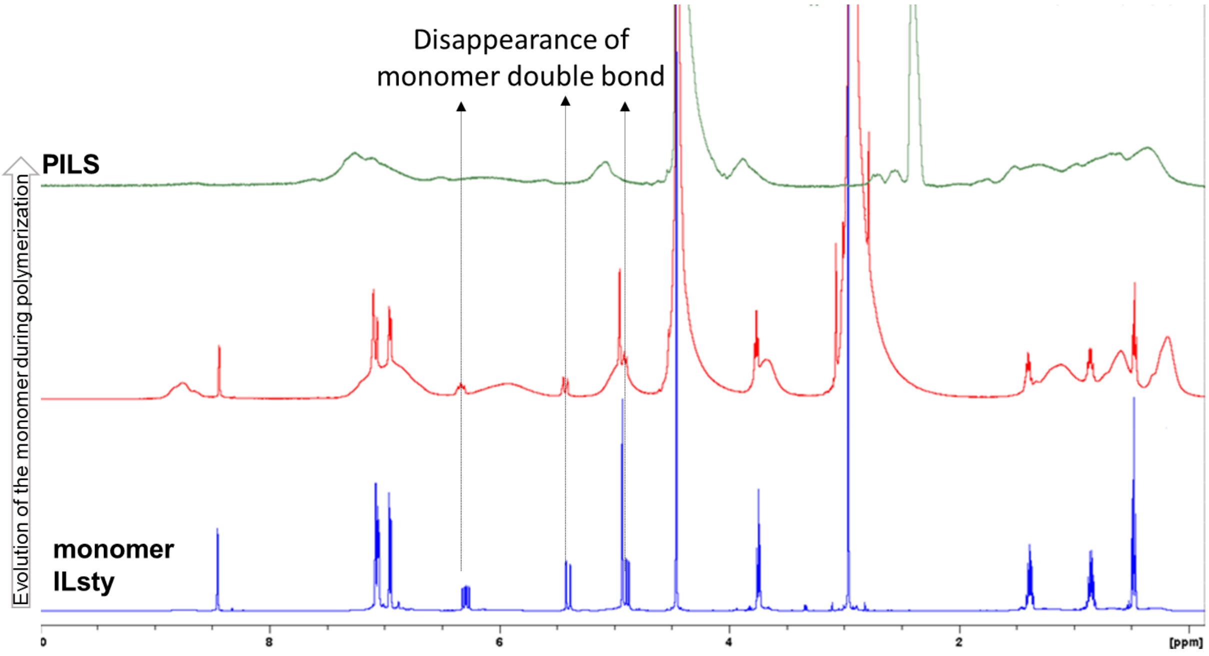

Figure S1. Evolution of ^{1}H NMR spectra (Bruker Avance III 500 MHz) during polymerization. Samples were dissolved in D_2O.
